# Short- and long-term effects from the communication between carbon-irradiated cancer cells and non-irradiated human cells: implication for radiotherapy and space radiation

**DOI:** 10.1093/jrr/rrt153

**Published:** 2014-03

**Authors:** Narongchai Autsavapromporn, Masao Suzuki, Cuihua Liu, Takeshi Murakami

**Affiliations:** 1 Research Program for the Application of Heavy Ions in Medical Sciences, Research Center for Charged Particle Therapy, National Institute of Radiological Sciences, 4-9-1, Anagawa, Inage-ku, Chiba-shi 268-3555, Japan; 2 Division of Therapeutic Radiology and Oncology, Department of Radiology, Faculty of Medicine, Chiang Mai University, 110 Intavaroros, Amphur Muang, Chiang Mai 50200, Thailand

**Keywords:** gap junction intercellular communication, bystander effect, genomic instability, heavy ion radiotherapy, space radiation

## Abstract

Heavy ions are one of the most effective treatment for cancer patient and the common types of radiation in space, such as carbon ions [ 1, 2]. However, the mechanism underlying the response between carbon-irradiated cancer cells and neighboring bystander normal cells remains unclear. Using the layered tissue culture strategy [ 3], human glioblastoma (T98G) cells were exposed to high linear energy transfer (LET) carbon ions (LET ∼76 keV/µm) with a single dose of 6 Gy or dose divided into three fractions given at consecutive day (2 Gy × 3 days) at Heavy Ion Medical Accelerator in Chiba. Within 10 min after irradiation, carbon-irradiated T98G cells were trypsinzied and co-cultured with human skin fibroblasts (NB1RGB) in the presence or absence of gap junction inhibitor (18-α-glycyrrhetinic: AGA). During this time, irradiated T98G cells and bystander NB1RGB cells were grown on either side of an insert with 3 µm pores. Following 4 h post-irradiation, bystander NB1RGB cells were then harvested and assayed for clonogenic survival and micronucleus (MN) formation or allowed to grow for 10 weeks and assayed for MN formation. Relative to control, both single- and fractionation irradiation showed that the bystander NB1RGB cells that co-cultured with carbon-irradiated T98G cells exhibited reduced cloning efficiency and associated with increased MN formation. In contrast, treatment with AGA showed a significant increase in survival and decrease in MN formation in bystander cells. These indicated that the role of gap junction intercellular communication (GJIC) in the propagation of bystander response from the communication between carbon-irradiated cancer cells and bystander normal cells. Furthermore, the progeny of bystander cells that were co-cultured with carbon-irradiated T98G cells for 10 weeks showed that there was induction of MN formation in both cases. Interestingly, the level of MN formation is reduced if there is the inhibition of GJIC. This supports that a role of GJIC in the propagation of stressful effects in the progeny of bystander cells. Our results provide the clear evidence that the bystander effect and genomic instability depend on intercellular communication and GJIC may be a critical mediator in the observed effects.
